# Supplementary figures and images for: Infectious viral shedding of SARS-CoV-2 Delta following vaccination: A longitudinal cohort study
Source: PLoS Pathog. 2022 Sep 12;18(9):e1010802. doi: 10.1371/journal.ppat.1010802 (PMC9499220; doi:10.1371/journal.ppat.1010802)

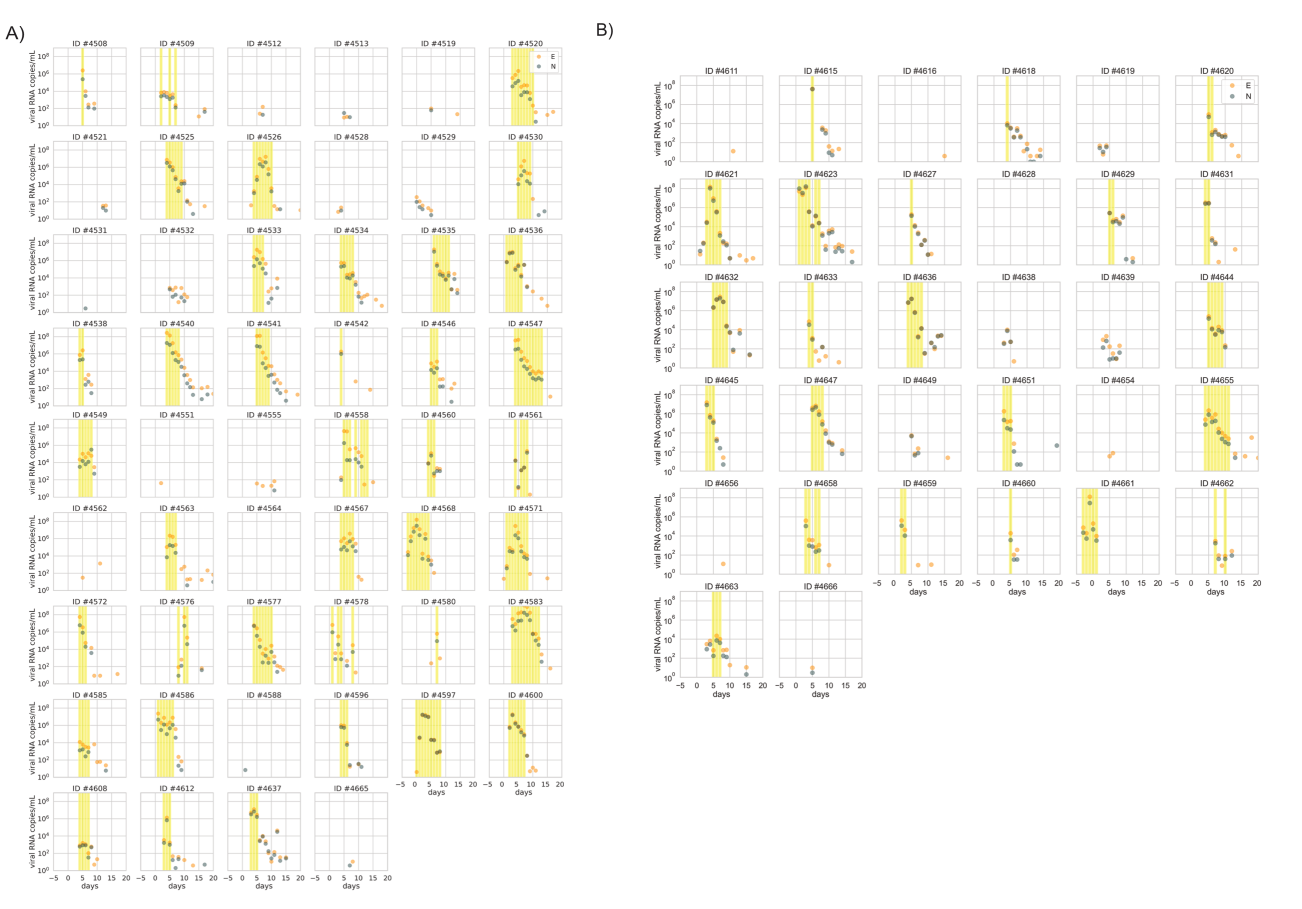

Supplement: S1 Fig — Scatter plots of each participant indicating the magnitude of viral RNA over time (days post symptom onset) and the period in which viral culture-positive samples were detected (yellow shaded area). Copies of N (grey) and E (orange) RNA targets are shown as dots in unvaccinated (A) and fully vaccinated (B) participants. (TIF) [file ppat.1010802.s001.tif]

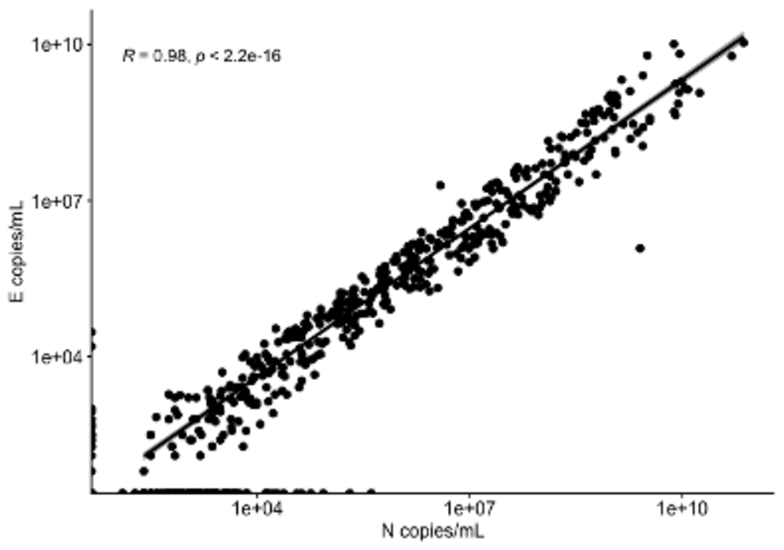

Supplement: S2 Fig — Nasal swabs collected from SARS-CoV-2 negative volunteers were added to viral transport media and spiked with SARS-CoV-2 WA1 strain or not spiked (NC). A) Copies of SARS-CoV-2 N were quantified by RT-qPCR following storage at room temperature (RT) and at 4°C for the indicated days and B) infectious titers were quantified by plaque assay following storage as indicated or following freeze-thaw (FT) cycles. (TIF) [file ppat.1010802.s002.tif]

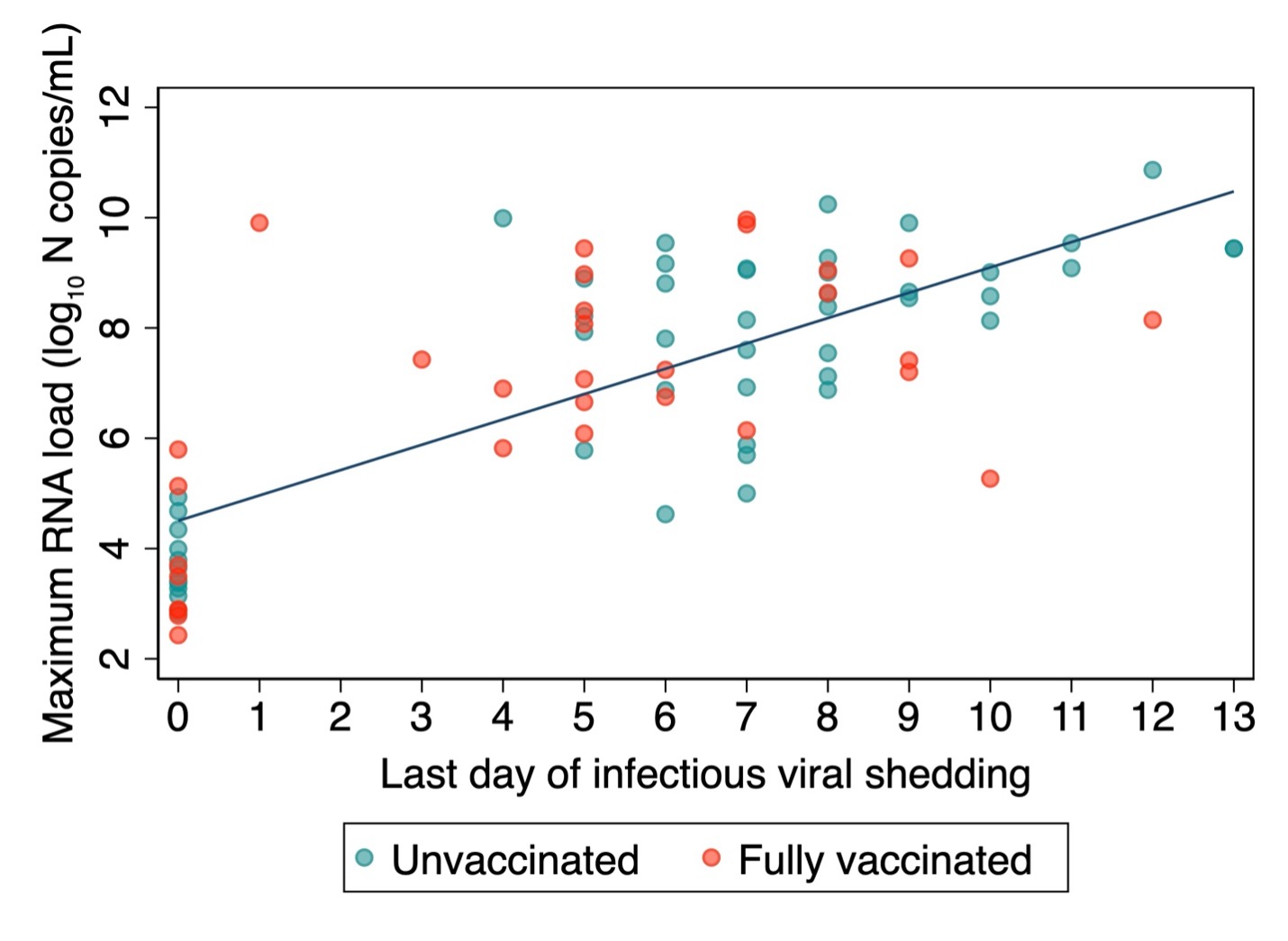

Supplement: S3 Fig — Correlation of the magnitude of N and E targets indicating the Pearson’s correlation coefficient. (TIF) [file ppat.1010802.s003.tif]

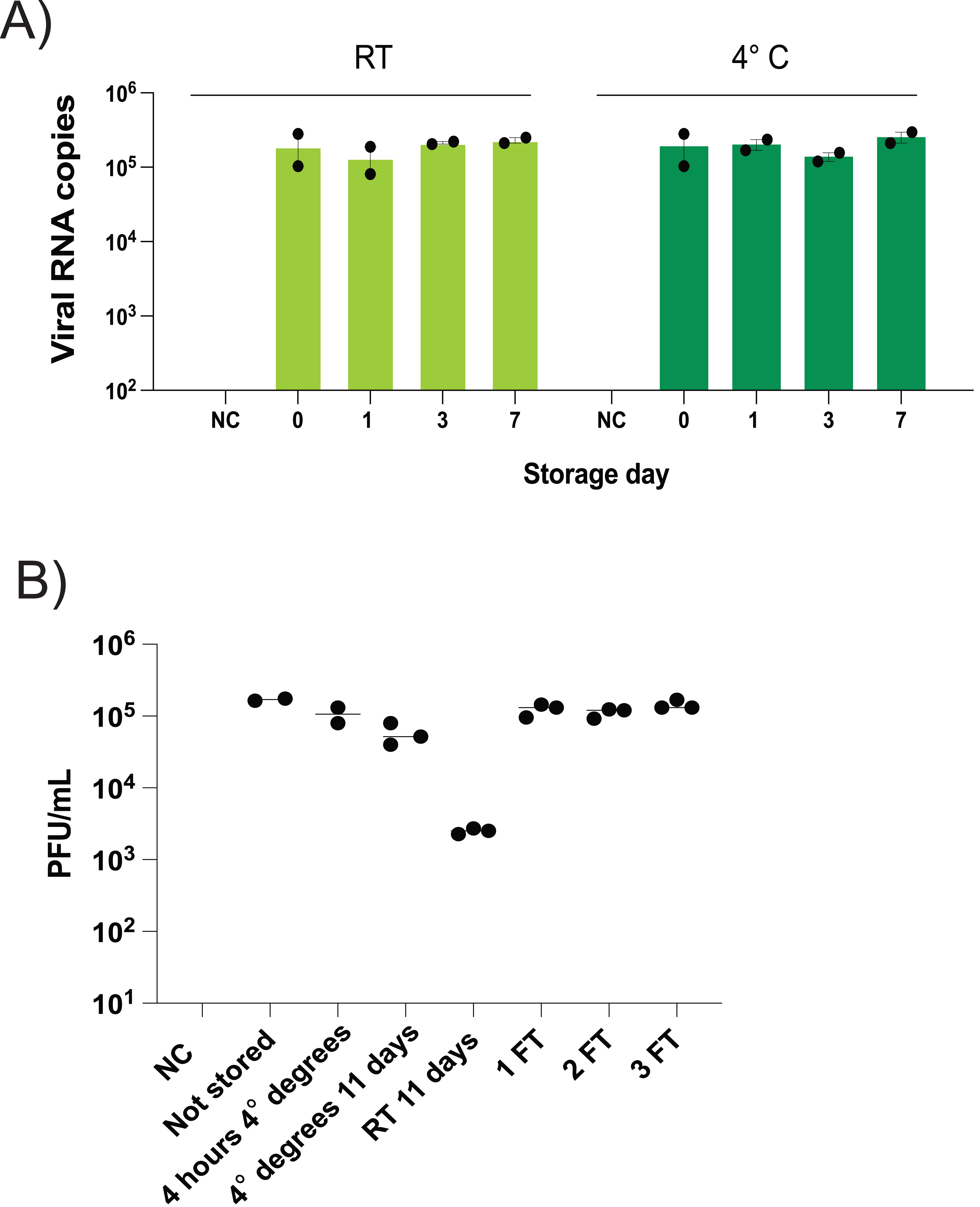

Supplement: S4 Fig — Correlation between the duration of infectious viral shedding and maximum RNA load showing the Pearson’s correlation coefficient. (TIF) [file ppat.1010802.s004.tif]
