# Supplementary material for: Infectious viral shedding of SARS-CoV-2 Delta following vaccination: A longitudinal cohort study
Source: PLoS Pathog. 2022 Sep 12;18(9):e1010802. doi: 10.1371/journal.ppat.1010802 (PMC9499220; doi:10.1371/journal.ppat.1010802)
Supplement: S1 Table — (DOCX) [file ppat.1010802.s005.docx]

**S1 Table. Oligonucleotide sequences for RT-qPCR**

|  | Forward primer | Probe | Reverse primer |
| --- | --- | --- | --- |
| SARS-CoV-2 N | GACCCCAAAATCAGCGAAAT | ACCCCGCATTACGTTTGGTGGACC | TCTGGTTACTGCCAGTTGAATCTG |
| SARS-CoV-2 E | ACAGGTACGTTAATAGTTAATAGCGT | ACACTAGCCATCCTTACTGCGCTTCG | ATATTGCAGCAGTACGCACACA |
| RNaseP | AGATTTGGACCTGCGAGCG | TTCTGACCTGAAGGCTCTGCGCG | CGGCTGTCTCCACAAGT |
