# Supplementary material for: Infectious viral shedding of SARS-CoV-2 Delta following vaccination: A longitudinal cohort study
Source: PLoS Pathog. 2022 Sep 12;18(9):e1010802. doi: 10.1371/journal.ppat.1010802 (PMC9499220; doi:10.1371/journal.ppat.1010802)
Supplement: S2 Table — (DOCX) [file ppat.1010802.s006.docx]

**S2 Table. Effect of vaccination type and time since vaccination on duration of viral shedding.**

| **Characteristic** | Overall duration of infectious viral shedding, median days (IQR) | P value^a^ |
| --- | --- | --- |
| **Type of mRNA vaccination** | | |
| mRNA-1273 (N=11) | 3 (0-5) | 0.13 |
| BNT162b2 (N=19) | 5 (0-8) |  |
| **Time from vaccination to infection** | | |
| <120 days (N= 21) | 5 (1-7) | 0.57 |
| >120 days (N=11) | 5 (0-7) |  |

^a^Mann-Whitney U test. IQR, interquartile range
