# Supplementary material for: Infectious viral shedding of SARS-CoV-2 Delta following vaccination: A longitudinal cohort study
Source: PLoS Pathog. 2022 Sep 12;18(9):e1010802. doi: 10.1371/journal.ppat.1010802 (PMC9499220; doi:10.1371/journal.ppat.1010802)
Supplement: Attachment — Submitted filename: ReviewersResponse.docx [file ppat.1010802.s007.docx]

Part II. Major issues.

Reviewer #2: 1- It would be very useful for authors to present clearly how many proportionally in the vaccinated vs unvaccinated had infectious virus post day 5 and day 10. This would help understand how many individuals are still potentially infectious. A recent study [https://www.ncbi.nlm.nih.gov/pmc/articles/PMC8996632/](https://urldefense.com/v3/__https:/www.ncbi.nlm.nih.gov/pmc/articles/PMC8996632/__;!!LQC6Cpwp!qkIsqaaLM-AZaO5fOIP4w4tRw8s3J5-MiSlKsBN7rqx1nNGYYnv1H0iNVcMTy-6dv-5AyafuX3cHEwFeqVGPTM2fpQ$) has shown that only 17% had a culture-positive virus post day 5.

Response: The reviewer has an excellent point here, particularly given the isolation guidance from CDC. We think it’s helpful to specifically state the proportion of individuals with infectious virus in the vaccinated groups at the suggested time points. We included this comparison after day 5 in table 2 in our initial submission and, in this revised manuscript, have added analysis after day 10 to table 2. We added this to our results section and discussion on lines 197 and 255.

2- There is also another confounder that was not addressed here, whether the unvaccinated had a previous infection. Although the effect of prior infection on infectiousness is not well studied, the immune response may suppress the infectious virus nevertheless. This should be noted as a limitation if this information is not available or included in the analysis.

Response: While we agree that it is important to consider previous infection, most unvaccinated individuals were recruited in the first year of the study when reinfections were uncommon and had only starting to be described as immune escape variants started to emerge and immunity started to wane (refs 33 and 34). We nevertheless measured IgG titers against N at recruitment in 14/52 unvaccinated individuals and found no evidence for previous infection in any unvaccinated participant as well as in any vaccinated participant (31/32 screened). We therefore consider previous infection not to have influenced our results. We have clarified this in the results (line 117) methods (line 356) and discussion (line 245).

3- Would it be possible to also look at the infectiousness duration among those vaccinated before 3 months vs after 3 months. Singanayagam et al. show that some of the vaccine's protective effects may start waning after 2-3 months, which may be why we are observing different results in different studies. The authors need to add this recently published paper: [https://www.nejm.org/doi/full/10.1056/NEJMc2202092](https://urldefense.com/v3/__https:/www.nejm.org/doi/full/10.1056/NEJMc2202092__;!!LQC6Cpwp!qkIsqaaLM-AZaO5fOIP4w4tRw8s3J5-MiSlKsBN7rqx1nNGYYnv1H0iNVcMTy-6dv-5AyafuX3cHEwFeqVHdtk4aNQ$) showing that most patients have culture-positive virus post day 5. But the difference between NEJM paper and CID paper [https://www.ncbi.nlm.nih.gov/pmc/articles/PMC8996632/](https://urldefense.com/v3/__https:/www.ncbi.nlm.nih.gov/pmc/articles/PMC8996632/__;!!LQC6Cpwp!qkIsqaaLM-AZaO5fOIP4w4tRw8s3J5-MiSlKsBN7rqx1nNGYYnv1H0iNVcMTy-6dv-5AyafuX3cHEwFeqVGPTM2fpQ$) and current analyses may be to do with host factors (age, comorbidities etc.) as well as specimen collection time, time from vaccination, and variant. This would be a good discussion point, in my view.

Response: In our original submission, we include an analysis (S2 table) of the duration of infectiousness in vaccinated individuals stratified into groups based on time since vaccination (under and after 4 months from vaccination to infection). We found no difference in the duration of infectiousness in these groups. Only 7 participants were infected within 3 months of vaccination, so using a 3-month cut off underpowered our analysis further. We have expanded our discussion to include the suggested papers and discuss possible reasons for the differences in or studies in the paragraph starting on line 254.

Reviewer #3: It would be beneficial to include the results of the experiments comparing the effect of freeze-thaw cycles on viral RNA and infectious virus quantification.

Response: As suggested by the reviewer, in this revised version we have included the results from our analyses relating to the effect of freeze-thaw cycles and storage conditions on viral RNA and infectious titers. We include these results in a new S2 figure and in the text on lines 134 and 172.

Part III. Minor issues

Reviewer #1.

1. Self-reported symptom onset can underly several biases e.g. recall bias. Thus, the authors might include some discussion on whether time of symptom onset is comparable between the groups infected with different viruses or vaccinated / unvaccinated individuals. E.g. Puhach et al 2022 shows that pre-VOC SARS-CoV-2 infections had lower infectious viral loads than Delta-infected unvaccinated individuals during the first 5 symptomatic days, which might also influence symptom onset.

Response: We acknowledge that self-reported symptom data may have underlying biases. We have expanded our discussion (paragraph starting on line 278) to include commentary on how these biases may have influenced how we defined days post symptom onset for patients and how infection with different viral lineages may have influenced this.

2. The findings presented here underline that vaccination reduced the likelihood of onward transmission from day 5 onwards, although from data presented in Figure 3B it looks like this also holds true for days 1-5? Figure 3 should be described in more detail in the figure legend. For Figure 3B, please elaborate on what exactly was compared and how the shown p-values were derived. It looks like for all days up to day 5 the proportion of viral culture + samples in the unvaccinated is higher than for the vaccinated, thus it is not clear whether the shown p-value refers to a comparison between d1 & day 5 among the fully vaccinated or comparing unvaccinated vs vaccinated overall. An improved image quality should be handed in for final submission.

Response: In the legend of figure 3B, we state that logistic regression was used to assess differences between vaccination groups, with analysis split into two bins spanning days 1-5 and 6-12. Even though over days 1-5 the proportion was lower in vaccinated participants, this did not reach statistical significance, likely due to the smaller number of specimens available for this analysis (indicated in the box in figure 3B). We go on to explain in detail how the logistic regression model was done in the methods section. We therefore expanded our description in the figure legend to clarify this.

4. It should be underlined that even as infectious virus can be isolated from patient’s samples there is no defined cut-off value for virus concentrations that would lead to actual transmission. Thus, the authors might want to include some guidance on how their findings would influence quarantine recommendations. However, these implications might be not directly transferable to the post-booster era especially in the context of emerging Omicron variants.

Response: This is an important point. We have expanded our discussion to comment on the relationship between the detection of infectious virus and transmission (line 268). We also include a reference to a publication of this cohort describing transmission events of which 19% occurred after 5 days.

6. Update Bibliography e.g. Puhach et al 2022 is now published in Nature Medicine

Response: Published preprints have been updated in references.

Reviewer #2: 1- Although detecting the infectious virus is important and somewhat can be used as an indicator to understand the infectiousness potential of the host, there is now evidence that viral load is also important in transmission. Detecting culture-positive virus late in infection when viral load is very low may not lead to transmission. So, I believe some of the comments in the discussion need to be softened to address this. This paper showed that peak viral load happens early in infection [https://pubmed.ncbi.nlm.nih.gov/33521734/](https://urldefense.com/v3/__https:/pubmed.ncbi.nlm.nih.gov/33521734/__;!!LQC6Cpwp!qkIsqaaLM-AZaO5fOIP4w4tRw8s3J5-MiSlKsBN7rqx1nNGYYnv1H0iNVcMTy-6dv-5AyafuX3cHEwFeqVG-0fo5CQ$), likely contributing to most of the transmission events. I am yet to see a report demonstrating onward transmission late in infection. For instance, this study [https://pubmed.ncbi.nlm.nih.gov/33530000/](https://urldefense.com/v3/__https:/pubmed.ncbi.nlm.nih.gov/33530000/__;!!LQC6Cpwp!qkIsqaaLM-AZaO5fOIP4w4tRw8s3J5-MiSlKsBN7rqx1nNGYYnv1H0iNVcMTy-6dv-5AyafuX3cHEwFeqVH7kwULqA$) showed that those with persistent RNA positivity had zero onward transmission to their contacts. Therefore, an infectious virus should not be equated to infectiousness as it depends on many factors. Also, important to address that the presence of an immune response will suppress the viable virus. So, detecting culture-positive virus in a vaccinated population may not be the same as detecting culture positive in an unvaccinated and naïve population.

Response: This is an important point raised by reviewer 1. We have expanded our discussion on lines 268 and 273 to address the points raised and softened some of the language. However, ~20% of transmission events in this cohort occurred after 5 days of symptom onset from the index case, so the probability of transmission later in the acute infectious period is still significant. We revised this point in the Discussion section to read as follows: “Despite epidemiological evidence that most transmission occurs early in the infectious period(42), our findings provide a biological rationale for why some degree of transmission still occurs in the late shedding period (beyond 5 but not after 10 days of symptoms) particularly in those with high peak viral loads (**Supp. Figure 3**)”

2- One of the caveats of the study, as also addressed briefly by the authors, is that the patients were included within 5 days after symptom onset. as they presented, 31% of unvaccinated individuals had very high Ct values, meaning they were already beyond the peak viral load. But, also there is a caveat that delta infections likely had an earlier peak so this study might have missed the peak in those individuals too. So, studies like this one [https://pubmed.ncbi.nlm.nih.gov/34756186/](https://urldefense.com/v3/__https:/pubmed.ncbi.nlm.nih.gov/34756186/__;!!LQC6Cpwp!qkIsqaaLM-AZaO5fOIP4w4tRw8s3J5-MiSlKsBN7rqx1nNGYYnv1H0iNVcMTy-6dv-5AyafuX3cHEwFeqVGhZfcHXw$) where contacts are recruited and followed up, will provide much better information about the peak viral load. So, I think the results of this study should be highlighted– the results are in line with the current analysis. However, they haven't investigated infectious virus; they had epidemiological evidence of onward transmission: "The mean viral load decline rate of the fully vaccinated delta group was also faster than those of the alpha group (pp=0·84) and the unvaccinated delta group (pp=0·85).

Response: We have expanded our discussion on the effect of delta peak VL in the paragraph starting on line 278 We discuss the findings of Singanayagam et al. extensively in the introduction and discussion sections.

3- I agree with the authors' conclusion that their analysis underestimates the true difference in infectiousness period between vaccinated and unvaccinated.

4- It's unclear why the authors used Vero-hACE2-TMPRSS2 cells for cell lines; more information is needed to support this approach. Was there a specific reason or rationale? It would be good to provide some background information about using these cell lines.

Response: We appreciate the opportunity to clarify our rationale for use of these cell lines. Vero cells stably expressing the protease TMPRSS2 were shown to enhance isolation of SARS-CoV-2 relative to Vero E6 cells. We reasoned that expression of human Ace2 would further enhance the sensitivity of this cell line to detect infectious SARS-CoV-2. This rationale and publication has been added to the methods section on line 394.

Reviewer #3: This paper would greatly benefit from an update literature search about longitudinal studies of infectious viral shedding. Several studies have been published that do not appear in the the references. Also, some of the referenced pre-prints might now be published.

Response: The reviewer has a good point, particularly as the COVID-19 literature grows rapidly. We updated the references we cite to account for recently published studies and add these to our discussion. References to preprints that are now published have been updated.
